# tDCS stimulation segregates words in the brain: evidence from aphasia

**DOI:** 10.3389/fnhum.2013.00269

**Published:** 2013-06-14

**Authors:** Valentina Fiori, Susanna Cipollari, Margherita Di Paola, Carmelina Razzano, Carlo Caltagirone, Paola Marangolo

**Affiliations:** ^1^Istituto Di Ricovero e Cura a Carattere Scientifico Fondazione Santa LuciaRoma, Italy; ^2^Dipartimento di Medicina Interna e Sanità Pubblica, Università de L'AquilaL'Aquila-Coppito, Italy; ^3^Department of Neurology, University of Tor VergataRoma, Italy; ^4^Department di Medicina, Facoltà di Medicina, Università Politecnica MarcheAncona, Italy

**Keywords:** tDCS, brain stimulation, aphasia rehabilitation, lexical deficits, language areas, word recovery

## Abstract

A number of studies have already shown that modulating cortical activity by means of transcranial direct current stimulation (tDCS) improves noun or verb naming in aphasic patients. However, it is not yet clear whether these effects are equally obtained through stimulation over the frontal or the temporal regions. In the present study, the same group of aphasic subjects participated in two randomized double-blind experiments involving two intensive language treatments for their noun and verb retrieval difficulties. During each training, each subject was treated with tDCS (20 min, 1 mA) over the left hemisphere in three different conditions: anodic tDCS over the temporal areas, anodic tDCS over the frontal areas, and sham stimulation, while they performed a noun and an action naming tasks. Each experimental condition was run in five consecutive daily sessions over three weeks with 6 days of intersession interval. The order of administration of the two language trainings was randomly assigned to all patients. Overall, with respect to the other two conditions, results showed a significant greater improvement in noun naming after stimulation over the temporal region, while verb naming recovered significantly better after stimulation of the frontal region. These improvements persisted at one month after the end of each treatment suggesting a long-term effect on recovery of the patients' noun and verb difficulties. These data clearly suggest that the mechanisms of recovery for naming can be segregated coupling tDCS with an intensive language training.

## Introduction

In these last years, a small but growing body of evidence have already indicated that non-invasive brain stimulation techniques, such as transcranial magnetic stimulation (TMS) (Naeser et al., 2005; Martin et al., 2009) and transcranial direct current stimulation (tDCS) (Monti et al., 2008, 2012; Baker et al., 2010; Fiori et al., 2011; Flöel et al., 2011; Fridriksson et al., 2011; Kang et al., 2011; Marangolo et al., 2013), can modulate the language system and, in particular, lexical retrieval. Although, most of these studies suggest that both techniques might be helpful in enhancing noun or verb naming, it is still an open question which stimulated language area might exert the greatest influence.

Some reports, using rTMS or tDCS in the healthy population, have already pointed to a crucial role of the temporal regions, and, in particular of the left Wernicke's area, in noun naming (Töpper et al., 1998; Mottaghy et al., 2006; Sparing et al., 2008). Töpper et al. (1998) have used rTMS to stimulate the left motor cortex, the left Wernicke's area and the right Wernicke's homologous area. A significant shortening of picture naming latencies was present only after stimulation over the left Wernicke's area. The same results were found by Mottaghy et al. (2006). Similarly, in a tDCS study, Sparing et al. (2008) comparing different stimulation conditions (anodic, cathodic, and sham stimulation over the left Wernicke's area and anodic stimulation of the homologous right Wernicke's area) in a group of 15 healthy subjects found faster responses only after anodic tDCS over the left Wernicke's area.

However, other studies have suggested a possible involvement in noun retrieval of the frontal region too. In a group of healthy subjects, Fertonani et al. (2010) found a facilitatory effect for noun naming after anodal tDCS stimulation over the DLPFC. More recently, Holland et al. (2011) targeted left frontal activity using 2 mA-tDCS during an fMRI study of overt spoken picture naming in 10 healthy volunteers. Each of the 107 pictures to be named was presented simultaneously with an auditory cue. Participants were instructed to name the object aloud as quickly and as accurately as possible. Faster naming responses in noun naming correlated with decreased blood oxygen level-dependent signal in Broca's area during the anodic tDCS over this area were found compared to sham stimulation.

To date, contradictory results have been reported also in the brain-damaged populations. Naeser et al. (2005) have shown that the application of slow rTMS suppressing the activation of the anterior portion of the right Broca's homolog (right pars triangularis), for 10 consecutive days, improved noun naming performance in four chronic, non-fluent aphasic subjects. Accordingly, in the same population, Kang et al. (2011) using cathodal tDCS stimulation over the right Broca's homolog area and concomitant noun-retrieval training demonstrated a significantly improved naming accuracy of treated items compared to sham stimulation. Similar results were obtained by Baker et al. (2010) and Monti et al. (2008) during application of anodal (Baker et al., 2010) and cathodal tDCS (Monti et al., 2008) over the left damaged frontal cortex. However, data have been reported where beneficial effects on noun naming during a concomitant language treatment resulted after anodal tDCS over the left temporal cortex (Fiori et al., 2011; Fridriksson et al., 2011; see also Flöel et al., 2011). Fridriksson et al. (2011) found beneficial effects after anodal tDCS over the left temporal cortex on vocal response time during a computerized anomia treatment in eight chronic aphasic participants. Similarly, Fiori et al. (2011) found that anodic tDCS stimulation over the left temporal region (including Wernicke's area) with concomitant language training for five consecutive days led to faster word retrieval in three aphasic patients at the end of treatment and three weeks later [see also Flöel et al. (2011)].

With regard to the recovery of verbs, Cotelli et al. (2006) assessed the effect of TMS applied to the left and right DLPFC on an object and action naming tasks in 15 patients with Alzheimer's disease (AD). In each subject, they found an improvement only for action (see also Cappa et al., 2002; Cotelli et al., 2012).

However, their results were not replicated in a subsequent study (Cotelli et al., 2008) in which rTMS applied to the DLPFC improved both noun and verb naming performance in AD patients not only in early, but also in a more advanced stage of their cognitive decline.

Until recently, only one report has specifically investigated tDCS influence in the improvement of verbs in the aphasic population (Marangolo et al., 2013). In this study, seven chronic subjects participated in an intensive language training for their action naming difficulties. During this training, each subject was treated with tDCS over the left hemisphere in three different conditions: anodic tDCS over the Wernicke's area, anodic tDCS over the Broca's area, and sham stimulation. In all patients, results showed a significantly better response accuracy only after anodic tDCS over the Broca's area.

In summary, the above mentioned studies seem to suggest that both rTMS and tDCS exert a positive influence in word retrieval. Nevertheless, it is still an open question which stimulated area might enhance the greatest effect. In most studies (Naeser et al., 2005; Martin et al., 2009; Baker et al., 2010; Fiori et al., 2011; Flöel et al., 2011; Fridriksson et al., 2011; Kang et al., 2011; see Holland and Crinion, 2012; Monti et al., 2012 for a review), the absence of a control condition through stimulation of another brain region did not allow to univocally attribute the effects to a specific contribution of the targeted region.

One way to resolve this issue is to compare the performance of the same aphasic population both in a noun and action naming task while stimulating different language areas.

This study was designed to investigate whether tDCS, over the frontal and the temporal regions coupled with an intensive language treatment, would differently improve noun and verb recovery in a group of seven participants with chronic aphasia.

## Materials and methods

### Participants

Seven aphasic subjects (5 men and 2 female) who had suffered a single left hemisphere stroke were included in the study. Inclusion criteria for the study were native Italian proficiency, pre-morbid right handedness, a single left hemispheric stroke at least 6 months prior to the investigation, and no acute or chronic neurological symptoms requiring medication.

The data analyzed in the current study were collected in accordance with the Helsinky Declaration and the Institutional Review Board of the IRCCS Fondazione Santa Lucia, Rome, Italy. Prior to participation, all patients signed informed consent forms.

### Clinical data

**Figure 1:**
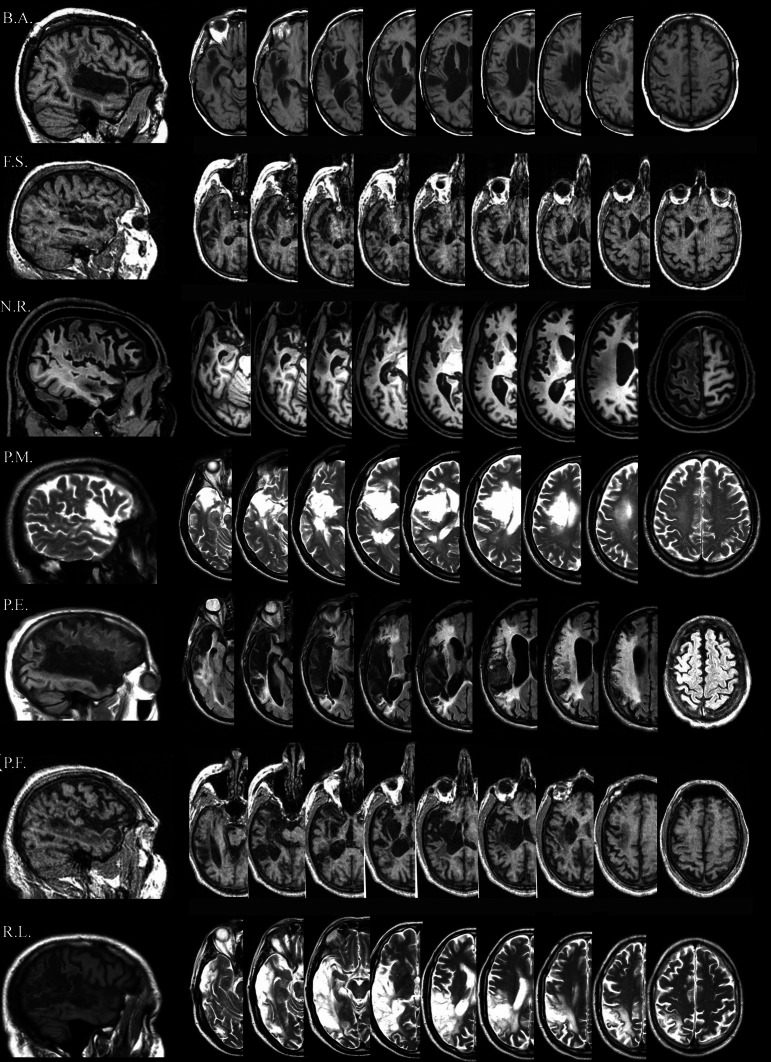
**Lesion descriptions for each aphasic patient. B.A.**'s lesion is localized in the left temporal cortex involving part of the temporal pole (superior part), the full extension of the superior temporal gyrus (Wernicke's area), and part of the middle temporal gyrus. A sufferance of cerebral white matter running in the angular gyrus and in the post central gyrus, with a relatively sparing of the corresponding parietal cortex is present. The lesion also involves the insula, although part of the insular cortex is spared. **F.S.**'s lesion is mainly localized in the left temporal-insular region. A further damaged area is at the level of the homolateral frontal lobe (mesial portion) involving the white matter running under the middle frontal gyrus. **N.R.**'s lesion is localized in the left fronto-temporo-parietal cortices. At frontal level, the damage laterally involves the inferior frontal gyrus (Broca's area), the middle frontal gyrus, the superior frontal gyrus and the precentral gyrus, and medially the medial frontal gyrus and the anterior cingulate gyrus. Posteriorly, the damage involves the temporal pole, the superior and the middle temporal gyrus, the post-central gyrus and the inferior parietal lobule. The lesion also includes the insula. **P.M.**'s lesion is localized in the left fronto-temporo cortices, involving the inferior frontal gyrus (Broca's area), and the temporal pole. The lesion also includes the insula. **P.E.**'s lesion is localized in the left fronto-temporo-parietal cortices, including the inferior frontal gyrus (Broca's area), the inferior part of the pre-central gyrus, the temporal pole, the full extension of the superior temporal gyrus (Wernicke's area), part of the middle temporal lobe, the inferior part of the post-central gyrus, the angular and part of supramarginal gyri. The lesion also includes the insula. **P.F**.'s lesion is mainly localized in the left fronto-temporal cortices, with a minor involvement of the homolateral parietal cortex. The damage includes the temporal pole, part of the superior temporal (Wernicke's area) and of the middle temporal gyri. The lesion also involves the insula. **R.L**'s lesion is localized in the left temporo-parieto-occipital cortices, including the temporal pole, the full extension of the superior temporal gyrus (Wernicke's area), part of the middle temporal lobe, the angular and the supramarginal gyri, the inferior parietal lobule and the superior occipital gyrus.

In all patients, the MRI revealed an ischemic lesion involving the left hemisphere (see Figure 1).

The aphasic disorders were assessed using standardized language tests [the Battery for the analysis of aphasic disorders, BADA test (Miceli et al., 1994); Token test (De Renzi and Vignolo, 1962)]. Subjects were also administered a Neuropsychological Battery (Orsini et al., 1987; Spinnler and Tognoni, 1987; Zimmermann and Fimm, 1994), which excluded the presence of attention and memory deficits that might have confounded the data (see Table 1).

**Table 1:** **Sociodemographic and Clinical data of the seven non-fluent aphasic subjects**.

**Subjects**	**Sex**	**Age**	**Ed. level**	**Time post-onset**	**Right Hemip**	**Right Hemian**	**Attentional Abilities (scores in percentile > 5 unimpaired)**	**Memory WM (cut/off 5 ± 2) STM (cut/off 7 ± 2) LTM (cut/off 5.5)**
							Alertness (tot): 76	WM: 4
B.A.	F	59	18	3 years and 3 months	+	−	Sustained Att (tot): 82	STM: 6
							Selective Att (tot): 54	LTM: 10
							Alertness (tot): 24	WM: 5
F.S.	F	71	5	1 year and 6 months	+	−	Sustained Att (tot): 21	STM: 6
							Selective Att (tot): 31	LTM: 10
							Alertness (tot): 58	WM: 4
N.R.	M	53	13	7 months	+	−	Sustained Att (tot): 73	STM: 6
							Selective Att (tot): 50	LTM: 10
							Alertness (tot): 88	WM: 5
P.M.	M	52	13	9 months	+	−	Sustained Att (tot): 50	STM: 5
							Selective Att (tot): 62	LTM: 11
							Alertness (tot): 18	WM: 4
P.E.	M	68	18	1 year and 8 months	+	−	Sustained Att (tot): 14	STM: 5
							Selective Att (tot): 16	LTM: 6
							Alertness (tot): 99	WM: 5
P.F.	M	44	13	7 years	+	−	Sustained Att (tot): 66	STM: 7
							Selective Att (tot): 84	LTM: 13
							Alertness (tot): 99	WM: 6
R.L.	M	62	11	4 years and 5 months	−	−	Sustained Att (tot): 58	STM: 6
							Selective Att (tot): 50	LTM: 13

*Results in the Neuropsychological Battery for Attention (Zimmermann and Fimm, 1994) and Memory deficits (Orsini et al., 1987; Spinnler and Tognoni, 1987) are also reported. Ed. Level, Educational Level; Right Hemip, Right Hemiparesis; Right Hemian, Right Hemianopia; WM, Working Memory; STM, Short-Term Memory; LTM, Long-Term Memory*.

The seven subjects were classified as non-fluent aphasics because of their reduced spontaneous speech with short sentences and frequent word-finding difficulties. They had no articulatory deficits with preserved word repetition and reading. In a naming task, all patients had lexical retrieval difficulties [BADA test (Miceli et al., 1994)].

### Materials

One hundred and two pictures of concrete nouns [i.e., box (*F* = 67, *L* = 7), pencil (*F* = 37, *L* = 6)] (Snodgrass and Vanderwart, 1980) and 102 videoclip of concrete actions [i.e., to shoot (*F* = 63, *L* = 7), to steal (*F* = 36, *L* = 6)] were used. Nouns and actions were matched for imageability (estimated on the basis of a sample of 21 normal participants along a seven-point scale), number of letters, age of acquisition [estimated on the basis of a sample of 20 normal participants along a nine-point scale; (Lotto et al., 2001)] and surface frequency [taken from De Mauro et al. (1993)]. Both imageability and age-of-acquisition ratings were collected by asking volunteers to judge printed words.

### Procedure

#### Transcranial direct current stimulation (tDCS)

tDCS was applied using a battery driven Eldith (neuroConn GmbH) Programmable Direct Current Stimulator with a pair of surface-soaked sponge electrodes (5 × 7 cm). A constant current of 1 mA intensity was applied on the skin for 20 min. If applied according to safety guidelines, tDCS is considered to be a safe brain stimulation technique with minor adverse effects (Poreisz et al., 2007). To stimulate the left temporal and frontal regions, two different electrode stimulation positions were used: the CP5 of the extended International 10–20 system for EEG electrode placement, which has been found to correspond best to the Wernicke's area (Oliveri et al., 1999; Fiori et al., 2011) and the F5 of the extended International 10-20 system for EEG electrode placement, which correspond best to the Broca's area (Nishitani et al., 2005; Naeser et al., 2010). In both conditions the reference electrode was placed over the contralateral frontopolar cortex (Nitsche and Paulus, 2000; Sparing et al., 2008).

**Figure 2:**
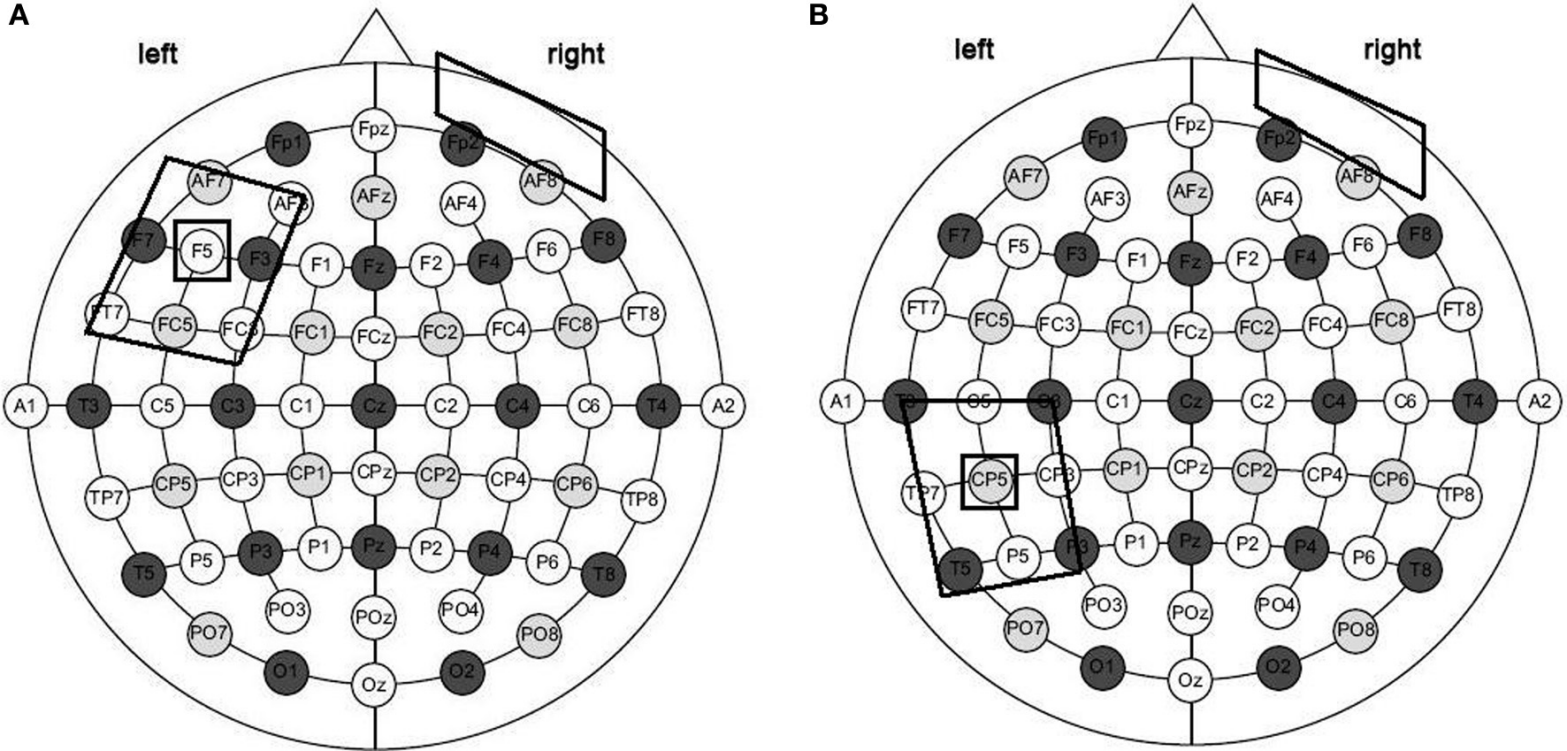
**Size and montage parameters of tDCS stimulation.** tDCS was applied using a pair of surface-soaked sponge electrodes of 5 × 7 cm. To stimulate the left temporal and frontal regions, two different electrode stimulation positions were used: the CP5 of the extended International 10–20 system for EEG electrode placement **(A)** and the F5 of the extended International 10–20 system for EEG electrode placement **(B)**. In both conditions the reference electrode was placed over the contralateral frontopolar cortex.

Overall, three different stimulation sessions were carried out: (1) anodic (CP5-A) stimulation of the left Wernicke's area; (2) anodic (F5-A) stimulation of the left Broca's area; and (3) sham stimulation over the Wernicke's area (CP5-S) for four out of seven patients and, for the remaining three, over the Broca's area (F5-S) (Figure 2). Sham stimulation was performed exactly like anodic stimulation over the left Wernicke's or Broca's area, but the stimulator was turned off after 30 s (Gandiga et al., 2006). To ensure the double-blind procedure, both the experimenter and the patients were blinded regarding the experimental and the sham conditions and the stimulator was turned on/off by another person.

For each subject, all pictures (*N* = 102) and actions (*N* = 102) were used. Stimuli belonging to each category were subdivided into three groups of 34 items each, matched for frequency, length, imaginability, and age of acquisition. For each category, one group of item was used for the left anodic Wernicke's stimulation, one for the left anodic Broca's stimulation and the third one for the sham condition. The assignment of each group of stimuli was randomized across conditions.

### Treatment

Once the electrodes had been placed on the scalp, the subjects performed the naming tasks while they received 20 min of tDCS. Three out of seven patients began with the noun naming treatment, while the remaining four started with the action naming training. The two treatments were separated by an interval of one month.

**Figure 3:**
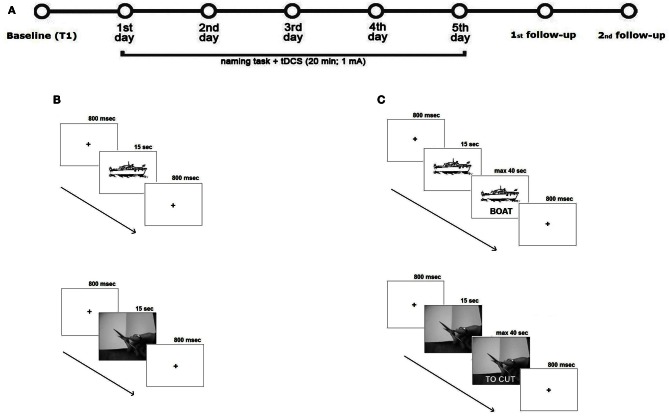
**Overview of study design. (A)** Subjects underwent two intensive language treatments for their noun and verb difficulties with 30 days of interval between the two trainings. During the two treatments, subjects received 20 min of tDCS. Each stimulation condition was performed in five consecutive daily sessions over three weeks, with six days of intersession interval. To measure baseline performance, at the beginning of each week and before the training each subject was asked to name the picture or the action videoclip, one at a time, without help. To measure the potential, long-term beneficial effects of tDCS two follow-up sessions were also carried 1 and 4 weeks after the end of each treatment condition. Subjects were asked to name aloud each picture or videoclip presented on the PC screen for 15 s and preceded by a fixation point, which lasted 800 ms. If the subject correctly named the item, the examiner manually recorded the response type on a separate sheet **(B)**. If the subject failed or did not answer within 15 s, the corresponding written name of the picture or of the action was presented below the image and the subject was asked to read the word aloud. The pair of stimuli remained on the screen until the subject read the word or 40 s elapsed **(C)**.

For each treatment, subjects were asked to name aloud each picture or videoclip that appeared on the PC screen (screen size 15″, viewing distance 1 m) for 15 s preceded by a fixation point, which lasted 800 ms (see also Raymer et al., 2006, 2007; Conroy and Scowcroft, 2012 for noun and action naming interventions). Only if the subject spontaneously correctly named the picture or the videoclip, the examiner manually recorded the response type on a separate sheet. If the subject failed or did not answer within 15 s, the corresponding written name was presented below the image and the subject was asked to read the word aloud. The subject never listen to the written word spoken aloud by the therapist. The pair of stimuli remained on the screen until the subject read the word or 40 s elapsed (see Figure 3). In any cases, subjects were not able to correctly read the word. For both trainings, each stimulation condition was performed in five consecutive daily sessions over three weeks, with six days of intersession interval. The order of items presentation was randomized across sessions. To measure baseline performance, at the beginning of each week and before the training each subject was asked to name the pictures or the videos, one at a time, without help. The order of conditions was randomized across subjects.

### Follow-UPS

At 1 and 4 weeks after each treatment, for each stimulation condition, all subjects were again shown the corresponding list of items and asked to name them without help. As before, the examiner manually recorded the answers.

### Data analysis

Data were analyzed with SPSS 13.0 software. Two different repeated-measures ANOVAs were applied on the mean percentage of response accuracy for nouns and verbs. We have excluded response time as a potential measure because we have found a large variability among patients. Three within-subject factors were included: *Task* [noun naming (NN) vs. verb naming (VN)], *Condition* (anodic Wernicke's area vs. anodic Broca's area vs. Sham) and *Time* [baseline (T1) vs. fifth training day (T5)] which, in the second analysis on the two follow-up sessions, was renamed *End-Post Treatment* factor [end of treatment (T5) vs. first follow-up (F1) vs. second follow-up (F2)]. Interactions were explored using the Scheffè *post-hoc* test.

## Results

The analysis showed a significant effect of *Time* [baseline (T1) vs. fifth training day (T5), *F*_(1, 6)_ = 160.04; *p* = 0.000]. Overall, subjects' performance significantly improved on the fifth day of training with respect to baseline [mean = 41%, SEM = 4 (T5) vs. mean = 21%, SEM = 3 (T1) *p* = 0.000]. Neither the *Task* [noun vs. verb naming, *F*_(1, 6)_ = 0.05; *p* = 0.831] nor the *Condition* [Wernicke's vs. Broca's area vs. Sham, *F*_(2, 12)_ = 2.37; *p* = 0.673] effects were significant.

The triple interaction *Time* × *Task* × *Condition* was also significant [*F*_(2, 12)_ = 60.36; *p* = 0.000]. The Scheffè *post-hoc* test revealed that the mean percentage of response accuracy for nouns and verbs significantly improved at the end of training in each condition with respect to baseline (differences between T5 vs. T1 for Wernicke's condition: 31%, *p* = 0.000, Broca's condition: 12%, *p* = 0.040, and Sham: 10%, *p* = 0.043 for nouns; differences between T5 vs. T1 for Broca's condition: 42%, *p* = 0.000, Wernicke's condition: 15%, *p* = 0.012, and Sham: 13%, *p* = 0.019 for verbs). However, although for both categories no significant differences emerged between the three conditions at baseline (differences between Wernicke vs. Broca = −2%, *p* = 1; differences between Wernicke vs. Sham = 3%, *p* = 0.999; differences between Broca vs. Sham = 5%, *p* = 0.918 for nouns; differences between Broca vs. Wernicke = −3%, *p* = 0.998; differences between Broca vs. Sham = −7%, *p* = 0.684; differences between Wernicke vs. Sham = −4%, *p* = 0.992 for verbs), at the end of training, there was a clear dissociation on the amount of improvement exerted by the temporal and the frontal stimulation in noun and verb recovery, respectively.

**Figure 4:**
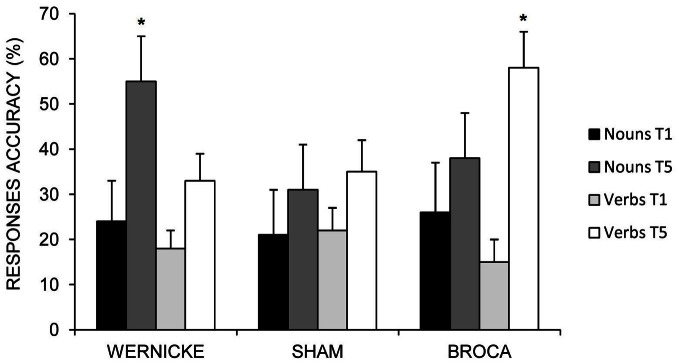
Mean percentage of correct nouns and verbs at baseline (T1) and at the end of treatment (T5) for the left Wernicke's, Broca's and the sham conditions (^*^0.000). Error bars represent standard error of the mean.

While the mean percentage of correct nouns was significantly greater in the Wernicke's condition with respect to the other two conditions (differences between Wernicke vs. Broca = 17%; *p* = 0.002; differences between Wernicke vs. Sham = 24%; *p* = 0.000; no significant differences between Sham vs. Broca = −7%; *p* = 0.571), the anodic Broca's condition determines the greatest improvement for verb naming with respect to the other two conditions (differences between Broca vs. Wernicke = 24%; *p* = 0.000; differences between Broca vs. Sham = 22%; *p* = 0.000; no significant differences between Sham vs. Wernicke = 2%; *p* = 0.999) (see Figure 4).

### Follow-UPS

The analysis showed a significant effect of C*ondition* [Wernicke's vs. Broca's area vs. Sham, *F*_(2, 12)_ = 6.86; *p* = 0.010] indicating a greater response accuracy for the Wernicke's and Broca's conditions with respect to sham [Wernicke's (mean = 40%, SEM = 3) vs. Broca's (mean = 41%, SEM = 4) vs. Sham condition (mean = 31%, SEM = 3) *p* = 0.010]. Neither the *Task* [*F*_(1, 6)_ = 0.00; *p* = 0.978] nor the *Time* effects [*F*_(2, 12)_ = 3.68; *p* = 0.057] were significant. The interaction *Task × Condition* was also significant [*F*_(2, 12)_ = 31.85; *p* = 0.000]. In agreement with previous data, the mean percentage of correct nouns was significantly greater in the Wernicke's condition than in the other two conditions [Wernicke's (mean = 51%, SEM = 5) vs. Broca's (mean = 32%, SEM = 6) vs. Sham conditions (mean = 30%, SEM = 5) *p* = 0.000; no significant differences between Broca's vs. Sham conditions, *p* = 0.997]. On the contrary, the Broca's stimulation exerted again the greatest influence in verbs' accuracy which was significantly higher in this condition than in the other two [Broca's (mean = 50%, SEM = 5) vs. Wernicke's (mean = 29%, SEM = 3) vs. Sham conditions (mean = 32%, SEM = 4) *p* = 0.000; no significant differences between Wernicke's vs. Sham conditions, *p* = 0.979].

**Figure 5:**
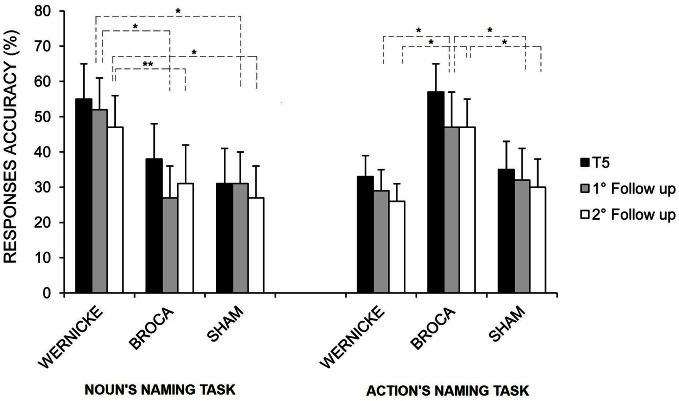
Mean percentage of correct nouns and verbs at the end of treatment (T5), at the first and second follow-ups for the left Wernicke's, Broca's, and sham conditions, respectively (^*^p = 0.00; ^**^p = 0.03). Error bars represent standard error of the mean.

Moreover, although no other interactions were significant, *post-hoc* analysis revealed that the greater amount in the mean percentage of correct nouns and verbs found for the Wernicke's and Broca's conditions, respectively, persisted both at the first and the second follow-ups (see Figure 5).

## Discussion

Our data suggest that the stimulation of different brain language regions differently affects the amount of improvement in noun and verb naming in a group of seven chronic aphasic patients.

Since previous studies have indicated that the best recovery is observed coupling tDCS with an intensive language treatment (Baker et al., 2010; Fridriksson et al., 2011; Marangolo et al., 2013), we chose to stimulate the patient during a picture naming task comparing the effects during anodic tDCS over the left frontal and temporal areas with a sham condition. In particular, when we analyzed the aphasic subjects' results, we found that all patients significantly recovered in each condition for both categories. This was due to the intensive language training that was performed daily during tDCS application (Bhogal et al., 2003). However, patients were much more accurate in nouns naming after the left temporal stimulation and in verb naming after the left frontal stimulation compared to the other two conditions which did not differ from each other in terms of response accuracy. These results allow us to affirm that the recovery in the two word categories was related to the stimulation of distinct brain regions. A further confirmation on the differential role played by the two regions comes from the follow-up testing which showed that the stimulation of the two language areas still exerted a different influence on the recovery of the two categories at one and four weeks after the end of each treatment.

As stated in the introduction, previous rTMS and tDCS reports with healthy and brain-damaged populations have already indicated a possible involvement of the temporal region in noun's naming (Töpper et al., 1998; Mottaghy et al., 2006; Sparing et al., 2008; Fiori et al., 2011; Flöel et al., 2011; Fridriksson et al., 2011) and the presence of a close relationship between the stimulation of the frontal region and the recovery of verbs (Cappa et al., 2002; Cotelli et al., 2006, 2008, 2012; Marangolo et al., 2013). However, while more consistent results were reported for verb retrieval, the data were less conclusive for nouns suggesting a possible interest of the frontal regions too (Monti et al., 2008; Baker et al., 2010; Fertonani et al., 2010; Holland et al., 2011; Kang et al., 2011). It might be argued that the facilitatory effect found by Holland et al. (2011) in noun naming after frontal stimulation was not specific to the process of word retrieval *per se* but was related to the activation of a concomitant automatic rehearsal process of the phonological word form exerted by target's spoken name (the auditory cue). In the same vein, it might be the case that in Naeser and Kang et al.'s studies (Naeser et al., 2005; Kang et al., 2011), the beneficial inhibitory influence exerted over the frontal right hemisphere has disengaged a more distributed left hemispheric network which did not necessarily restrict the recovery from nouns to a contribution of the left frontal cortex.

In our opinion, one way to disentangle the above contradictions is to compare within the same population, the performance of the patients in the two naming tasks while stimulating different language regions, as we did in the present study. We believe that our data clearly pointed to the presence of a close relationship between the stimulated region and the amount of recovery found for nouns and verbs. Results from transfer of treatment effects confirmed this hypothesis. Indeed, at the language tests, in six out of seven patients, there was a significant improvement in noun naming only after stimulation of the temporal region, while the ability to produce verbs significantly increased in five patients only after stimulation of the frontal region. Moreover, after frontal stimulation, some patients showed a significant improvement also in word repetition (N.R., P.E., R.L.) and reading (B.A., P.M., R.L.) while one patient (F.S.) had a significant recovery in writing under dictation task (see Table 2). It is widely known that the premotor region, including Broca's area, supports the rehearsal process necessary for refreshing the word memory trace during word repetition (Romero et al., 2006; Trost and Gruber, 2012). It has also been suggested that the premotor cortices play some role in implementing the activity patterns involved in reading and writing (Anderson et al., 1990; Lubrano et al., 2004). In our patients, the hypothesis that could be advanced is that the stimulation of the frontal region co-activated the surrounding premotor areas which lead to an improvement in other language tasks.

**Table 2:** **Number of correct responses at baseline, post-Broca, post-sham, and post-Wernicke administration of the standardized language tasks (BADA and Token test)**.

**Subj**	**Conditions**	**Oral noun naming**	**Written noun naming**	**Noun comp**	**Oral verb naming**	**Written verb naming**	**Verb comp**	**Word reading**	**Word writing under dictation**	**Word repetition**	**Token test**
B.A.	Baseline	15/30	0/22		16/28	0/22		10/92	0/46		16/36
	post -Broca (1)	16/30	0/22	40/40	17/28	0/22	20/20	34/92^*^	0/46	45/45	17/36
	post-Sham (2)	15/30	0/22		18/28	0/22		10/92	0/46		17/36
	post -Wernicke (3)	26/30^*^	0/22		18/28	0/22		15/92	0/46		25/36
F.S	Baseline	12/30	10/22		14/28	5/22			37/46		22/36
	post-Broca (2)	14/30	12/22	40/40	21/28^**^	10/22	20/20	92/92	46/46^**^	45/45	22/36
	post-Sham (1)	12/30	10/22		12/28	5/22			39/46		20/36
	post-Wernicke (3)	27/30^*^	13/22		20/28	7/22			32/46		26/36
N.R.	Baseline	0/30	0/22		0/28	0/22		10/92	0/46	25/45	9/36
	post-Broca (3)	9/30	0/22	40/40	10/28^**^	0/22	20/20	10/92	0/46	36/45^**^	8/36
	post-Sham (2)	9/30	0/22		2/28	0/22		15/92	0/46	30/45	9/36
	post-Wernicke (1)	12/30^*^	0/22		3/28	0/22		9/92	0/46	36/45	15/36
P.M.	Baseline	0/30	0/22		0/28	0/22		10/92	0/46		11/36
	post-Broca (2)	8/30	2/22	40/40	6/28^**^	0/22	20/20	25/92^*^	5/46	45/45	12/36
	post-Sham (3)	10/30	0/22		5/28	0/22		13/92	3/46		13/36
	post-Wernicke (1)	14/30^*^	1/22		0/28	0/22		15/92	5/46		13/36
P.E.	Baseline	0/30	0/22		0/28	0/22		10/92	0/46	13/45	4/36
	post-Broca (2)	5/30	0/22	40/40	15/28^**^	0/22	20/20	14/92	0/46	26/45^**^	6/36
	post-Sham (1)	0/30	0/22		5/28	0/22		15/92	0/46	13/45	4/36
	post-Wernicke (3)	13/30^**^	0/22		14/28	0/22		10/92	0/46	23/45	10/36
P.F.	Baseline	13/30	0/22		15/28	0/22		10/92	0/46		14/36
	post-Broca (1)	21/30	0/22	40/40	15/28	0/22	20/20	11/92	0/46	45/45	17/36
	post-Sham (3)	17/30	0/22		15/28	0/22		12/92	0/46		15/36
	post-Wernicke (2)	17/30	0/22		21/28	0/22		10/92	0/46		17/36
R.L.	Baseline	11/30	0/22		14/28	0/22		55/92	0/46	2/45	10/36
	post-Broca (3)	19/30	0/22	40/40	25/28^*^	0/22	20/20	74/92^**^	0/46	12/45^**^	10/36
	post-Sham (1)	11/30	0/22		11/28	0/22		55/92	0/46	1/45	9/36
	post-Wernicke (2)	20/30^**^	0/22		11/28	0/22		60/92	0/46	2/45	11/36

The order of conditions (indicated by the number in brackets) was randomized across subjects. For five out of seven subjects, χ^2^(^*^) test indicated a significant difference before and after the Broca's condition in verb naming and, for six out of seven subjects, before and after the Wernicke's condition in noun naming (

* < 0.0001;

** * < 0.05)*.

*Subj, Subjects; Comp, Comprehension*.

Although the neural mechanisms responsible for tDCS stimulation are still not known, some authors have affirmed that anodic stimulation elicits an extended increase in cortical excitability probably due to depolarization of neuronal membrane and to changes in the synaptic connections of the *N*-methyl-D-aspartate (NMDA) receptors involved in long-term potentiation (Nitsche and Paulus, 2000). This happens when the stimulated area is supposed to be directly involved in the investigated language task. However, others (Naeser et al., 2005; Kang et al., 2011) found an improvement also after stimulating through rTMS (Naeser et al., 2005) or tDCS (Kang et al., 2011) the right language homologous areas.

To date, the role played by the right hemisphere in language recovery is more controversial than that of the left regions. Some evidence has suggested that the right activity may support recovery only if homotopic areas take over the function of the lesioned left language areas (Saur et al., 2006, 2008; Turkeltaub et al., 2011) even if they are computationally less efficient. Alternatively, the right hemisphere may limit recovery if its processing is dysfunctional, or if transcallosal projections from the right inhibit the left language areas (Naeser et al., 2005; Martin et al., 2009; Kang et al., 2011). Most of the studies have affirmed that the quality of improvement is dependent on the amount of spared neural tissue in the left hemisphere and, to a lesser extent, on the homologous areas in the right-hemisphere (Heiss and Thiel, 2006). It seems likely that the reactivation of undamaged network areas of the left hemisphere usually leads to a better outcome than the involvement of homotopic contra-lateral regions (Heiss and Thiel, 2006). For this reason, in our study, we choose to stimulate the left hemisphere regions as also suggested by previous tDCS studies which indicated that the stimulation of perilesional spared language areas close to the stimulation site in chronic aphasic patients enhances functional improvement (Baker et al., 2010; Fiori et al., 2011; Fridriksson et al., 2011; Marangolo et al., 2013).

Although some studies have shown that it is difficult to predict the distribution of the current (Baker et al., 2010), others have suggested that during naming the current is distributed around the stimulated region (Holland et al., 2011). The same results were found in an fMRI studies to measure tDCS effects during the stimulation of the motor cortex (Antal et al., 2011, 2012). Since our patients had very different left hemispheric lesions we reasoned that in this way we have targeted the two regions. Considering that all of our patients had left-hemisphere damage, it might be argued that the better recovery observed after the left temporal and frontal stimulation, respectively, for nouns and verbs, was not related to the stimulated areas but to a greater sparing of one of the two region.

However, as shown in Figure 1, all of our patients had very different left cerebral stroke which in some cases predominantly involved the posterior (B.A., P.F., and R.L.) or the anterior areas (P.M.) and in others either completely damaged the Wernicke's and Broca's area (N.R., and P.E.) or totally spared the two stimulated regions (F.S.).

We believe that independently of the amount of left spared cerebral tissue, the different improvement found for the two word categories clearly indicate at least a partial segregation of the beneficial effects into a specific brain region. It might be the case that, in our patients, tDCS has enhanced the capacity of the left-hemisphere spared areas close to the stimulated region to make compensatory plastic changes resulting in improved performance.

We are aware that the present approach, due to the small sample used and to the lack of functional magnetic resonance imaging data, does not allows to draw firm conclusions about the underlying neural mechanisms by which tDCS affected subjects' performance.

However, overall, it consents to define some important points about language rehabilitation in persons with aphasia. Indeed, it confirms several previous reports that highlight the importance of coupling tDCS with the naming treatment. Moreover, it clearly suggests the importance to apply different stimulation protocols to the aphasic populations to enhance the best recovery.

### Conflict of interest statement

The authors declare that the research was conducted in the absence of any commercial or financial relationships that could be construed as a potential conflict of interest.
